# Exploring the Readiness for Digital Health Literacy Transformation and Intervention Preferences From the Perspectives of Patients With Cancer, Caregivers, and Health Care Professionals: Qualitative Interview Study

**DOI:** 10.2196/77738

**Published:** 2026-03-04

**Authors:** Hind Mohamed, Turki Alanzi, Jon Salsberg, Mudathir Mohamed, Dervla Kelly

**Affiliations:** 1 Faculty of Education and Health Sciences, School of Medicine, University of Limerick, Henry Street, Limerick, V94 T9PX, Ireland; 2 Health Information Management and Technology Department, Imam Abdulrahman Bin Faisal University, Dammam, Saudi Arabia; 3 Emergency Medicine Department, E1 Health Cluster, King Fahad Specialist Hospital, Dammam, Saudi Arabia

**Keywords:** digital health, eHealth, internet, mobile health, mHealth, digital health literacy, intervention, training, microlearning, microlearning-based, Saudi Arabia

## Abstract

**Background:**

Technology is changing the way the world communicates and how we learn, remember, and transform information. The ascendancy of the internet has dramatically altered the landscape of health information access and seeking behaviors. This transformation is embodied by the concept of digital health literacy (DHL) and the need for interventions that improve DHL.

**Objective:**

This study aims to explore readiness for DHL transformation and intervention preferences from the perspectives of patients with cancer, caregivers, and health care professionals.

**Methods:**

We conducted semistructured telephone and on-site interviews with 19 patients with cancer, 6 caregivers, and 10 oncology health care professionals. Purposive sampling was used to recruit the participants. We followed the 7 stages of the Framework Method analysis: transcription, familiarization with the interview, coding, developing a working analytical framework, applying the analytical framework, charting the data into the framework matrix, and interpreting the data. This was used to investigate participants’ beliefs about technology adoption, their preferences for DHL training, and the facilitating conditions for adopting such training. We used a hybrid deductive-inductive approach to data analysis, starting with a priori themes and allowing emergent themes to develop as the analysis progressed. The Unified Theory of Acceptance and Use of Technology informed our data generation and analysis.

**Results:**

The following 6 themes emerged from the analysis: introducing technology-driven solutions, simplifying technology training materials, providing user-friendly training materials, patient-centered care, partnership, and addressing cultural and linguistic barriers. We found that patients with cancer and caregivers were self-sufficient and motivated to use new technology to improve their DHL; however, health care professionals were concerned about the reliability of online information. By mapping interview themes to the Unified Theory of Acceptance and Use of Technology, we identified specific recommendations for the creation of a DHL intervention: content should be concise, easy to understand, and web-based; content should include training on how to identify reliable cancer information; patients would like to be involved in content creation in addition to medical and government stakeholders; and content should be accessible in multiple languages.

**Conclusions:**

Patients with cancer and their caregivers were motivated to use modern technology to improve cancer DHL, despite the quality issues raised by health care professionals. The participants’ preferences regarding DHL training align with the innovative microlearning pedagogy. Microlearning could leverage technology to deliver tailored DHL training for patients with cancer. Collaboration with multiple medical and nonmedical stakeholders could facilitate the delivery of cancer DHL training. Future work should focus on designing and assessing the feasibility of implementing a microlearning-based DHL training program that involves end users and diverse stakeholders.

## Introduction

Online technologies have changed societies and affect our daily lives in many ways. This is reflected in the growing use of the internet and mobile devices by consumers to access health information and services [1]. Saudi Arabia’s Vision 2030 outlines an ambitious plan to transform the health care sector through digitalization [2]. This transformation includes aspects of digital health literacy and technology-driven health engagement, focusing on improving access, efficiency, and patient empowerment through digital health [3].

In Saudi Arabia, approximately two-thirds or more of individuals get their health-related information from social media or the internet [4]. Internet use can be particularly effective when there are barriers to accessing traditional health information, especially for stigmatized conditions such as cancer [5,6]. Patients with cancer widely seek digital health information, as demonstrated in a study reporting that 76.4% of survivors of cancer use the internet for health-related information [7].

Digital health is now considered a complement to cancer care to meet key unmet needs, such as “support to help cope with a diagnosis” and “cancer education and information” [8]. Indeed, one study reported “information and education” as one of the most needed digital health functions for patients with cancer, caregivers, and health care providers [9].

Digital health literacy (DHL) is the ability to use information and communication technologies to find, evaluate, create, and communicate health information, and it requires both cognitive and technical skills [10]. Information searching is often described as a problem-solving task that involves a series of complex cognitive processes, such as inferences and decision-making [10]. Complex or confusing digital health information is especially difficult for patients with cancer since their cognitive abilities decrease due to the effects of the cancer and chemotherapy treatment [11]. A 2023 systematic scoping review found that patients with cancer and caregivers struggle to identify reliable information resources and can be misled or misinterpret information online [12]. A study in Saudi Arabia demonstrated that participants with higher DHL scores were more likely to have increased breast cancer literacy [13]. Another study reported that almost one-third of participants face challenges formulating precise health-related inquiries, highlighting the critical need for specialized DHL education initiatives in Saudi Arabia [14]. Mackert et al [15] and Zarcadoolas et al [16] have advocated for user-friendly educational interventions to bridge the DHL gap.

Health education needs to adapt and develop innovative ways of relating appropriately to the way we live, work, and learn today. A recent systematic review reported many interventions to improve laypeople’s DHL, including interactive workshops, community outreach programs, and educational initiatives. However, no one has used a novel educational approach or targeted patients with cancer [17]. Low DHL in cancer care contributes to adverse health outcomes, hinders self-management of patients’ conditions, and is considered a barrier to engaging with digital health technologies [18].

Digital health readiness is defined as a patient’s ability and comfort with using digital tools for health care engagement [19]. This concept expands on existing literature describing DHL to include factors such as health care trust, acceptance of technology, and relevance to care. Current approaches to DHL intervention design are evolving from viewing patients as merely passive consumers of care to viewing them as active coproducers in their care management [20,21]. Coproduction in health care delivery is a reciprocal, collaborative process in which patients and health care providers work together to deliver care, sharing information and setting goals to create value [20]. Digital health interventions in cancer care have been reported to reduce symptoms [22], improve health behaviors [23], and promote psychological well-being and quality of life [24].

Nutbeam [25] outlined the need to invest in research to improve our understanding of how effective health interventions should be implemented. Eccles et al [26] further argued that we need to see greater use of theoretical approaches underpinning the design and implementation of interventions. Although theories have been used to evaluate digital health interventions [27], there is a lack of theoretically informed research in the field of digital health readiness.

The Unified Theory of Acceptance and Use of Technology (UTAUT) is a comprehensive digital health readiness framework that integrates and extends various theories to explain the acceptance and use of technology [28]. It incorporates elements from 8 previous models of technology acceptance, namely the Theory of Reasoned Action, the Technology Acceptance Model, the Motivational Model, the Theory of Planned Behavior, a combined model of Technology Acceptance Model and Theory of Planned Behavior, the Model of Personal Computer utilization, the Diffusion of Innovations theory, and Social Cognitive Theory [28]. It has 4 determinants (performance expectancy, effort expectancy, social influence, and facilitating conditions) and 4 moderators (gender, age, experience, and voluntariness of use) [28]. The UTAUT model has demonstrated its broad applicability across diverse fields, including the Internet of Things [29], artificial intelligence products [30], and electronic health technology [31,32], but has not yet been applied to educational interventions targeting patients with cancer.

The aim of the paper was to explore the readiness for DHL transformation and intervention preferences from the perspectives of patients with cancer, caregivers, and health care professionals using the UTAUT model. To our knowledge, our paper is the first to use the UTAUT to explore readiness for DHL transformation and intervention preferences from the perspectives of patients with cancer, caregivers, and health care professionals. The UTAUT was chosen to help us understand the following objectives: participants’ beliefs about technology adoption, preferences regarding DHL training adoption, and the conditions that facilitate DHL training adoption. Knowledge of technology adoption is crucial to ensure the delivery of quality cancer care education.

## Methods

The COREQ (Consolidated Criteria for Reporting Qualitative Research) checklist [33] was used to guide reporting of the study (Checklist 1). It also provides a structured account of the study’s methodological rigor, transparency, and researcher reflexivity.

### Study Design

The study design domain describes a qualitative content analysis approach [34] informed by the UTAUT. The UTAUT was relevant because it enables in-depth exploration of the unconscious behaviors that drive the uptake of health education initiatives. Semistructured on-site and telephone interviews were conducted with patients with cancer attending the oncology department for chemotherapy treatment and their caregivers, as well as oncology health care professionals at King Fahad Specialist Hospital (KFSH), Dammam, Saudi Arabia. The researchers selected the semistructured interview format because it allowed them to preformulate questions, compare data across participants, and adjust questions as necessary during the interview [35].

### Sampling and Recruitment

Purposive sampling was used to provide rich, relevant, and diverse data that were not intended to be representative of a broader population [36]. We deliberately sampled a mix of ages, genders, and socioeconomic statuses from different participant types, including patients with cancer and their caregivers and oncology health care professionals, based on preidentified inclusion and exclusion criteria (Textbox 1). Patients and caregivers were recruited face to face. Paper-based posters, recruitment emails, and telephone calls were also used to facilitate the recruitment. Oncology health care professionals were recruited through gatekeepers in the oncology department. We recruited 44 participants, including 24 patients, 6 caregivers, and 14 health care professionals; 5 patients and 4 health care professionals declined to participate. In total, 35 participants agreed to participate in the study. Saturation was used as a guiding principle to assess the adequacy of the purposive sample [37].

Textbox 1. **Inclusion and exclusion criteria for the different participant types, including people with cancer, their caregivers, and health care professionals involved in cancer care.**

Inclusion criteria

1. Patients with a personal history of cancer who are considered in remission
2. Family/friend caregivers involved in helping care for patients with cancer for 2 or more hours per week, providing emotional or other support
3. Health care professionals involved in cancer care (assistant consultants/consultants, nurses, health educators)
4. ≥18 years old, male or female, and able to understand and answer in Arabic
5. Must have used the internet to search for cancer information

Exclusion criteria

1. Patients with no personal history of cancer; patients with a personal history of cancer but not considered in remission
2. Caregivers with no significant involvement in helping with care as defined by less than 2 hours per week of emotional or other support
3. Health care professionals not involved in cancer care
4. < 18 years old , unable to understand and answer in Arabic
5. Never used the internet to search for cancer information

### Interview Question Design

A panel of 5 stakeholders agreed on the interview questions. The panel included 2 clinicians and 3 academics with expertise in DHL. The semistructured interview questions were open-ended focusing on the following areas: (1) digital learnability, (2) DHL training mode of delivery, (3) DHL training information content and display, (4) DHL training interval and duration, (5) DHL training design, and (6) facilitators of the DHL training adoption. The questionnaire initially had 10 questions. An additional question was added following a small number of pilot interviews to better understand the facilitators for adopting DHL training. This question and the DHL training design question received the most interest from patients with cancer and caregivers. Health care professionals were most interested in discussing the DHL training content; however, differences emerged among patients with cancer, their caregivers, and health care professionals’ preferences. We developed the interview guide in English to allow discussion with the English-speaking researchers. The interview guide was translated into Arabic, as the interviews were conducted in Arabic per the protocol and inclusion criteria. Participants were provided with an Arabic version of the topic guide before consultation. Multimedia Appendix 1 includes the English and Arabic versions of the interview guide. Sociodemographic data were collected via a brief pre-interview questionnaire that was posed by the interviewer and answered by the participants.

### Data Collection

A female PhD researcher (HM) with no prior interaction with participants carried out the interviews in Arabic. No one else was present besides the participants and the researcher. The researcher disclosed that the study was part of her doctoral research on DHL. The researcher has professional-level fluency in Arabic and English and is a DHL expert, with experience in qualitative research, the research topic, and Saudi culture. The researcher completed the Good Clinical Practice course before conducting the study. The researcher conducted 35 telephone and on-site (KFSH) interviews with patients with cancer, caregivers, and oncology health care professionals following written and verbal informed consent. The interviews were conducted in cycles between August 15, 2024, and February 15, 2025, at times and locations convenient to the participants. Interviews were audio-recorded, transcribed, and coded using a thematic analysis approach within the Framework Method described in the following paragraphs [38]. No field notes were made during and/or after the interview. No repeat interviews were carried out. At the close of each interview, the audio recordings were transcribed into Microsoft Word. The description of the coding tree was not included in the main manuscript nor its appendices but can be shared upon reasonable request. Most of the interviews lasted 30 minutes to 45 minutes. However, interviews with patients with cancer and caregivers lasted between 30 minutes and 35 minutes, while health care professionals ’ interviews lasted longer (35‐45 min). There were no differences in interview quality between patients with cancer or caregivers and health care professionals or between telephone and on-site interviews. We reached data saturation after conducting the 35 interviews, as no new themes were emerging during interviews 34 and 35. We first assessed code saturation to ensure it was sufficient to understand the issues identified. Second, we assessed meaning saturation to ensure that no further dimensions, nuances, nor insights into the issues could be found. We also evaluated whether specific characteristics of codes influenced code or meaning saturation to provide parameters for estimating saturation based on the nature of codes developed in a study [37]. These approaches provided confidence that the collected data adequately captured the breadth of relevant perspectives within the study population, thereby ensuring the depth and credibility of the findings.

### Data Analysis

Audio recordings of the interviews were made with the participants’ consent and transcribed verbatim in Arabic. The quotes were translated forward and backward into English by the principal investigator (HM; researcher translator). A bilingual health literacy expert consultant reviewed the translation (MM). The transcriptions were analyzed by iteratively reading transcripts. Thematic analysis was conducted using the Arabic versions of the transcripts; the results section used English translations of the quotations for readability by a wider audience. We used a hybrid deductive-inductive approach to data analysis [38], starting with a priori themes and allowing emergent themes to develop as the analysis progressed. This blend helped balance theoretical grounding with a rich, data-driven understanding of the topic. Given the potential value of theory as a heuristic for qualitative analysis processes [39], data generation and analysis were informed by the UTAUT. Emergent themes were subsequently mapped onto the 4 determinants of the UTAUT [28]. The mapping process relied on iteratively moving between the emergent themes and the UTAUT determinants to build knowledge about applying UTAUT to the project data.

The Framework Method sits within a broad family of analysis methods often termed thematic analysis or qualitative content analysis. The Framework Method can be adapted for use with qualitative analyses that are deductive, inductive, or combined [40]. It also facilitated constant comparative techniques by reviewing data across the matrix; as such, it was found to be appropriate for our paper as it involves different types of participants (patients, caregivers, and health care professionals) [40]. The following 7 stages of the Framework Method analysis were followed: transcription, familiarization with the interview, coding, developing a working analytical framework, applying the analytical framework, charting the data into the framework matrix, and interpreting the data [40].

Reflexivity measures were applied to mitigate potential bias arising from the researcher’s background in DHL. Two researchers from different professional backgrounds independently conducted coding in NVivo 14 to explore the development of inductive themes and the application of deductive ones. This approach helped to ensure intercoder reliability and to heighten reflexivity [41].

Transcript analysis was carried out at primary and secondary levels. Whereas first-level coding created descriptive, low-inference codes directly from the data, second-level analysis allowed for the reorganization of primary codes into appropriate categories, subthemes, and themes [41].

Member checking is crucial in qualitative research to ensure credibility and validity, reduce researcher bias, increase trustworthiness of the findings, and ensure participants’ expertise was fully used [42]. A researcher prepared a draft of the findings, including anonymous quotations, 1 month after data collection and shared it with one-half of the participants individually (an equal number of health care professionals and patients). The researcher asked questions about accuracy, interpretation, completeness, fairness and respect, and clarification. The participants provided their answers through written feedback. The feedback was used to revise interpretations, clarify ambiguities, and add missing information.

To ensure the awareness of reflexivity in that one’s own beliefs can affect interpretation [41], 3 additional members of the research team, a cancer survivor, and a consultant oncology doctor, reviewed the themes, subthemes, and categories and refined them, seeking clarity on language and suggesting additional work. The research team worked in a group throughout the analysis, comparing coding, discussing how the data related to the UTAUT coding frame, and refining the coding frame. Data analysis clinics were also used to strengthen the initial inductive analysis and to map themes onto the UTAUT. A narrative description was provided for each of the themes speaking to readiness for DHL transformation and intervention preferences.

### Ethical Considerations

All the procedures performed in the study involved human participants. Ethical approval was obtained before data collection from the KFSH research ethics committee (April 23, 2024/EXT0429). The method of collecting and analyzing data was carried out in a manner that was compliant with all applicable ethical norms. Full disclosure was made regarding the investigation’s objective and the legal rights of those participating. Each participant was given a pseudonym to preserve confidentiality and maintain anonymity. Every participant provided informed consent before the interview began. Participation in the study was entirely voluntary throughout the process. Participants did not receive compensation for their participation in the study. Data will be stored on the principal investigator’s (HM) password-protected Outlook OneDrive account for 2 years then destroyed.

## Results

### Participants’ Characteristics

The first 24 patients who met the study criteria were invited to participate in the study; 5 patients declined (3 men and 2 women) because they were too ill to be interviewed on the day of the appointment. In addition, 6 caregivers agreed to participate in the study. Of the 14 health care professionals recruited, 4 refused to participate (3 nurses and 1 health educator) because they were too busy during the data collection period. Only 10 health care professionals (assistant consultants and consultants) agreed to participate in the study. The total number of patients, caregivers, and health care professionals interviewed was 35. Table 1 presents the participants’ characteristics including age, gender, and participant category. Questions were asked regarding demographics, including education, and socioeconomic status; however, many participants declined to disclose these, most likely as they were considered sensitive information. Given the extent of the missing data, these variables have not been reported.

**Table 1.** Participant characteristics for those who participated (n=35) in the semistructured interviews in King Fahad Specialist Hospital, Dammam, Saudi Arabia, between August 15, 2024, and February 15, 2025.

Participant category	Participants, n	Male, n	Female, n	Age (years), range
Patients with cancer	19	9	10	30‐65
Cancer caregivers	6	1	5	30-50
Oncology health care professionals	10	7	3	40-55

### Qualitative Findings

Findings on readiness for the DHL transformation and intervention preferences are presented in Textbox 2 and framed around the 4 determinants of the UTAUT. We identified 6 themes and 9 subthemes under the UTAUT determinants. For the first 2 subthemes, there were categories that were considered individually as indicated in the following sections.

Textbox 2. **The 4 determinants of the Unified Theory of Acceptance and Use of Technology and their definitions, themes, subthemes, and categories describing the factors that influence digital health literacy training preferences in people with cancer in a sample size of 35 participants.**

Performance expectancy (user’s belief that using technology will enhance their search performance and productivity)

1. Introduction of technology-driven solutions
  - Digital literacy learnability
    1. Self-efficacy for using the technology to search for cancer information
    2. Willingness to engage with new technology to improve cancer digital health literacy
  - Demand for technology-driven solutions to improve cancer digital health literacy
    1. Addressing the digital divide
    2. Designing an educational digital health literacy training

Effort expectancy (the degree of ease associated with the use of technology)

1. Simplifying technology training materials
  - Bite-sized information
  - Desire for reliable information
2. Providing user friendly training materials
  - Self-paced training
  - Short-duration training

Social influence (the impact of external factors on an individual’s decision to adopt technology)

1. Patient-centered care
  - Willingness to be involved in the design of the training material

Facilitating conditions (availability of resources, support, and infrastructure necessary for technology adoption and use)

1. Partnership
  - Collaboration with multiple stakeholders (medical and nonmedical)
2. Addressing cultural and linguistic barriers
  - Providing bilingual training material

### Performance Expectancy

There were 2 subthemes under performance expectancy: digital literacy learnability and demand for technology-driven solutions to improve cancer DHL.

#### Digital Literacy Learnability

Digital literacy learnability refers to the self-efficacy of patients with cancer and their willingness to engage with technology.

Patients with cancer and their caregivers spoke about their self-efficacy in navigating online cancer information. Inversely, many health care professionals expressed concerns about the quality of cancer information retrieved by patients online.

I feel confident when opening web browsers to search for cancer information.

[Patient 5]

I feel confident to click on a link to visit a cancer website.

[Caregiver 2]

Nowadays, patients are increasingly dependent on the internet to search for cancer information; however, most of the information they brought to me was unreliable.

[Health care professional 3]

Patients with cancer and caregivers expressed being motivated to engage with new technologies, with a view to learning and improving their cancer care. Health care professionals also noted patients’ eagerness to search online, often before their doctor’s visit.

I am interested to learn about my health through the new technology channels.

[Patient 8]

I believe learning through the new technology can affect patients' health positively.

[Caregiver 5]

Most of the patients nowadays prefer searching online even before visiting the doctor.

[Health care professional 7]

#### Demand for Technology-Driven Solutions to Improve Cancer DHL

Demand for technology-driven solutions to improve cancer DHL refers to addressing the digital divide to ensure that technology is accessible to all users, regardless of their geographical location or socioeconomic status and designing educational DHL training.

Patients, caregivers, and health care professionals reported a preference for web-based DHL training. They believed that online training would help address the digital divide and facilitate digital inclusion.

Going far away from my home to get the training is costly, and I don't have the energy like before.

[Patient 5]

I won't be able to attend training conducted even 5 minutes away from my house, as it requires a lot of dedication.

[Caregiver 4]

I would say if the educational training is provided online, it will be easily accessible for the consumers and more engaging.

[Health care professional 2]

Patients and caregivers spoke about designing a smartphone app to deliver the DHL training. On the other hand, many health care professionals demonstrated a need for an educational video to improve cancer DHL.

I feel more engaged with smartphone applications as I hold my phone most of the day.

[Patient 15]

If the educational materials are provided through the applications, they will be easily accessible.

[Caregiver 6]

I believe designing an educational video to be available for the general public would be of great value, as they provide easy-to-catch information.

[Health care professional 7]

#### Effort Expectancy

There were 4 subthemes under effort expectancy: bite-sized information, desire for reliable information, self-paced training, and short-duration training.

##### Bite-Sized Information

Patients with cancer and caregivers emphasized the importance of providing concise educational content to reduce cognitive effort and time. Health care providers discussed the impact of chemotherapy on the learning abilities of patients with cancer, necessitating the provision of small-bundle information.

I am a busy person with my family, work, and now a cancer patient; I don’t have that energy like before to learn complex educational materials.

[Patient 11]

I think providing educational material in small bites may reduce the learning burden.

[Caregiver 1]

Patients with cancer face many difficulties concerning learning, especially after receiving chemotherapy; they would benefit from small pieces or guides of information.

[Health care professional 8]

##### Desire for Reliable Information

Patients and caregivers expressed a need for training on identifying reliable cancer information. They recommended learning tips for evaluating the reliability of online information and identifying high-quality websites, while health care providers spoke about the importance of educating laypeople about common cancer misconceptions.

I want to learn about excellent quality websites for lay people to search cancer information.

[Patient 13]

I want to learn how to evaluate the reliability of online health information.

[Caregiver 1]

Many patients with cancer come to my clinic with worsening symptoms and poor prognosis due to misinformation. It is essential to increase public awareness about common cancer misconceptions.

[Health care professional 3]

##### Self-Paced Training

Most patients and caregivers stated that they like the flexibility of online training and being able to go at their own pace.

Accessing the training material with no fixed schedule and the ability to set my times would help me to engage.

[Patient 12]

I want to reach the training when I want, at the time I want, and with the time I have.

[Caregiver 6]

##### Short-Duration Training

Patients, caregivers, and health care professionals spoke about providing short-duration training to reduce cognitive load and optimize attention span for memorizing information.

It could even be an educational training that does not exceed 10 minutes to be able to focus and memorize it.

[Patient 11]

They can be 10 minutes, but then they are almost too long.

[Caregiver 6]

Short training, 5 minutes maximum to reduce the learning load.

[Health care professional 8]

### Social Influence

There was 1 subtheme under social influence: willingness to be involved in the design of the DHL training.

Patients with cancer expressed a strong desire to be involved in the design of the training material.

Caregivers and health care professionals believed that the patient voice should be heard to ensure relevance and acceptability of training materials.

Nobody knows it better than yourselves, having gone through it… You can set up all the processes on paper: This happens, this happens, this happens, but until you actually press play and go through it.

[Patient 6]

People who set the training materials have never passed through a cancer journey. They see it from the other side of the desk, but to actually involve people who have been on the receiving end.

[Caregiver 15]

Getting patients with cancer involved could help to cover a huge gap that comes between the delivery and acceptance of the training materials.

[Health care professional 6]

### Facilitating Conditions

There were 2 subthemes under facilitating conditions: collaboration with multiple stakeholders (medical and nonmedical) and providing bilingual training materials.

#### Collaboration With Multiple Stakeholders (Medical and Nonmedical)

Patients with cancer, caregivers, and health care professionals spoke about the essential role of collaborative work in the design of the training materials. They emphasized the role of technology assistants, governmental authorities, and health care professionals in designing the educational materials.

I trust the oncology doctors, nurses, health educators, researchers, and governmental authorities. Why don't they put their hands together to deliver such training?

[Patient 3]

It is not an easy game that one person can play; it is the health of cancer patients… it needs different expertise in different sectors. It is worth connecting to deliver such a lifesaving game.

[Caregiver 1]

I would see it as a cooperative work; one hand will not clap. We are not in front of the computer to see how this work can be done. We want to work as one unit to see this work delivered properly.

[Health care professional 1]

#### Providing Bilingual Training Materials

Patients with cancer, caregivers, and health care providers expressed a need for providing bilingual DHL training to deliver culturally appropriate material.

I think it would be worthwhile if the training is provided in Arabic and English.

[Patient 19]

Our native language in Saudi Arabia is Arabic, while some residents from different cultures speak English also. I believe providing an educational training in both languages would be useful for both.

[Caregiver 4]

Providing bilingual training in both Arabic and English may facilitate the use of the educational training by the end consumers from different cultures.

[Health care professional 6]

**Figure 1.**
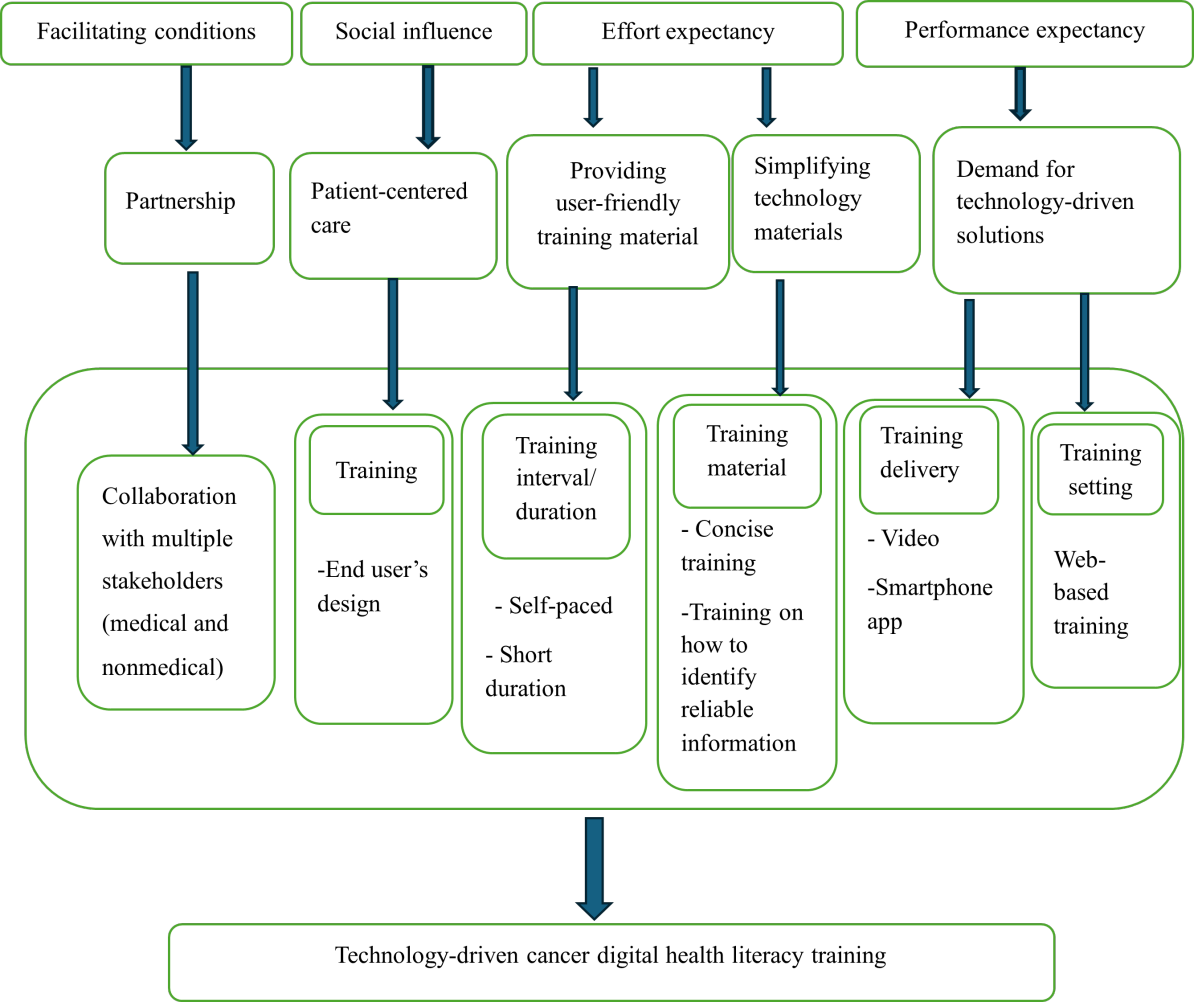
Cancer digital health literacy conceptual framework based on the main findings of the study in conjunction with the Unified Theory of Acceptance and Use of Technology, including the useful training components derived from the thematic analysis and theory determinants.

We integrated the findings into the commonly identified themes to generate a conceptual framework for cancer DHL training (Figure 1). The framework explores how the pre-identified themes and subthemes were mapped onto the 4 determinants of the UTAUT to generate technology-driven cancer DHL training.

## Discussion

### Principal Findings

We found that patients with cancer and caregivers were self-sufficient and motivated to engage with new technology to improve cancer DHL; however, health care professionals were concerned about the reliability of online information. We identified specific recommendations for creating cancer DHL training: Content should be concise, easy to understand, web-based, and delivered through technology-driven solutions; content should include training on how to identify reliable cancer information; patients would like to be involved in content creation; and training should be self-paced and of a short duration. Our findings demonstrated collaboration between medical and nonmedical stakeholders to facilitate DHL training adoption.

### Comparison With Prior Work

#### Participants’ Beliefs About Technology Adoption

Our findings indicated that patients with cancer and caregivers were self-sufficient and motivated to use novel technology to improve cancer health literacy. Previous studies in Saudi Arabia reported that most participants prefer to use the internet to search for health information [43-45]. A qualitative focus group study found that most participants recognized the benefits of using technology to manage their health care, despite expressing concerns about its use [16].

Car et al [46] identified several barriers to the use of digital health information, including a lack of motivation and cognitive skills to find and use information. The expectation that online health information will be useful is one of the strongest predictors of internet use [47], and a lack of skills was debated as one possible reason for not using the internet [46]. Specifically, potential users may lack the skills to effectively locate, evaluate, and use online health information [48]. Motivation was reported as an essential step in line with education for DHL training adoption [46].

#### Participants’ Preferences Considering DHL Training Adoption

Our findings indicated that most participants preferred web-based DHL training. This approach has been reported in previous DHL interventions [49-53]; however, those interventions were not microlearning-based. An online learning environment has certain limitations; as such, learning materials should be as explicit and purposeful as possible, thus microdesigned. Microlearning could leverage the use of technology to deliver tailored DHL training but not a technology-dependent format of learning [54].

Our findings indicated that the participants preferred to receive DHL training through technology-driven solutions, namely smartphone apps and videos. A study in Saudi Arabia used an educational video to improve participants’ ability to detect health-related misinformation in WhatsApp messages [55]. Nokes and Reyes [56] targeted patients with HIV through an educational video about identifying reliable internet sites. As a cost-effective approach, mobile health apps can also appropriately deliver interventions to patients with cancer [57] and patients with chronic conditions [58]. The “SUCCESS app” is a DHL cross-platform application developed to support Australian adults with chronic kidney failure to improve patients’ critical health literacy skills [59]. The “WebChoice” application was developed to provide reliable and useful links and resources for patients with cancer [60].

Effective microdesigned videos and health apps have recently been reported in health professional education studies [61-63]. However, to our knowledge, no microdesigned DHL apps or videos targeting patients with cancer have been developed yet. More research is needed to understand participants’ views on how smartphone apps and videos could be microdesigned to improve DHL for patients with cancer.

We noted that the preferred style for information display for patients with cancer was a bite-sized format. Buchem and Hamelmann [64] contended that large bundles of information on the internet are often ignored, whereas small pieces of the whole are tagged and linked in ways that create new patterns, ideas, and meaning. Microlearning, therefore, has the capacity to present information in the most common way people learn today [65]. The individual learning units had a clear, focused message that reinforced two of the aims of microlearning: reducing cognitive load on working memory [66] and adapting to the pressures of a limited attention span [67].

Our paper identified demand for training on how to identify reliable cancer information, including learning to identify high-quality cancer websites and tips for evaluating online information. Both approaches were observed in prior DHL interventions and were reported to be effective in a systematic review [17]. A DHL workshop introduced participants to cancer websites of various top-level domains and web pages accredited by the Health on the Net Foundation [68]. A DHL tutorial also introduced participants to the MedlinePlus.gov website to search for health information [69]. Previous interventions have used several tools to teach people how to evaluate online information, including the SEEK (Source, Evidence, Explanation, and Knowledge) criteria [70], the DISCERN critical appraisal tool [49], and the PILOT (purpose, information, links, originator, timeliness) criteria [71]. However, Bernstam et al [72] reported that commonly cited website quality criteria are not effective at identifying inaccurate online information about breast cancer. Future research could focus on using the quality criteria to assess their effectiveness in evaluating other cancer web pages.

Health care professionals in our study identified a need to increase public awareness of common cancer misconceptions. A cross-sectional study conducted in Saudi Arabia explored the 7 commonly identified cancer misconceptions in Saudi Arabia [45]. However, the results of the study are not available in lay language. The next essential step is translating and disseminating the research findings into lay language, which could be facilitated through technology-driven DHL interventions.

Our findings indicated that the participants preferred self-paced, short-duration DHL training. This approach was seen in prior DHL interventions [56,70]. However, none of those interventions fit within the microlearning time frame. Unlike formal learning, the time spent on microlearning ranges from a few seconds to 15 minutes [64]. With microlearning’s ability to space out the learning process over time, a more extended period is often required to learn the content, thereby leveraging retrieval spacing effects to improve information retention and support perpetual, sustainable lifelong learning [65].

Our findings indicated that cancer patients were interested in being involved in the design of cancer DHL training. Mohamed et al [17] reported using this approach in a few studies and considered it a facilitator of intervention use by end consumers. Kemp et al [18] called for using participatory design principles to address DHL in cancer care. Microlearning focuses on users’ social interactions to drive content creation and can transform consumers into producers (prosumers) [64]. We identified a small body of literature focusing on users’ feedback in the design of microlearning interventions in cancer care [73]. Future research could focus on developing microlearning-based DHL interventions from end users’ perspectives.

Several educational approaches have been trialed to improve consumers’ DHL, such as didactic lessons [71,74] and collaborative learning [75]. However, driven by the patient-centered care concept and the era of rapidly advancing technologies, Lee et al [76] called for the use of student-centered pedagogical approaches to educate health consumers. One such innovative pedagogy that could meet our participants’ demand for bite-size, discrete learning bundles is microlearning [77].

#### Facilitating Conditions for DHL Training Adoption

In our study, the participants called for a collaborative approach between medical and nonmedical staff to deliver DHL training. Previous research reported the delivery of several DHL interventions through the collaboration of multiple stakeholders [53,56,68,74]. Most of those interventions were reported to be effective in a systematic review study [17]. Partnerships have become increasingly relevant in the delivery of health care services over the last few decades [78]. For instance, a 2012 Institute of Medicine report listed calls for enhanced collaboration among the public, private, health care, and non-health care sectors to improve chronic disease prevention [79]. Other studies have echoed recommendations for cross‐sector collaboration to address social determinants of health [80,81]. Recently, the infosphere has been recognized by Morley et al [82] as a social determinant of health, defining it as the entire information environment that influences health-related behaviors and outcomes, alongside socioeconomic status, income, education, age, race, ethnicity, and gender.

### Strengths and Limitations

This study has several limitations. We only explored the perspectives of patients with cancer, caregivers, and oncology health care professionals. Our paper did not consider the opinions of policymakers, technology advisers, and other health care professionals (nurses and health educators). Future research can include diverse stakeholder types. Only 6 caregivers were included in our study. Future research could include more caregivers to compare their experiences with those of patients with cancer. The study focused on participants from a single hospital, limiting generalizability to broader populations. Future research should include a diverse sample across multiple health care settings. The study participants were all deliberate internet users, selected to understand their experiences with online navigation needs. Addressing noninternet users and considering their preferences for DHL intervention were not considered in our paper. Implementing digital health in cancer care must address the variability of DHL in recipients to enhance digital inclusion and digital health equity. The Framework Method used for thematic analysis was time-consuming and resource-intensive and requires a high level of training to be used successfully within a multidisciplinary team.

The UTAUT was found to be a good fit for our study, as it captured the content discussed in the interviews and helped explore which technology-driven DHL training works well from the perspectives of end users and health care providers. To our knowledge, our study is the first to apply the UTAUT to propose a microlearning-based DHL training program. However, we did not examine the effect of the UTAUT moderators (age, gender, experiences, voluntariness of use) on participants’ intention to adopt technology. Future research could use a health literacy lens to examine the interactions among those factors and participants’ technology adoption.

### Implications for Practice

To ensure patient-centered care, it is essential to involve the end users in designing future DHL interventions. Collaborating with multiple stakeholders across relevant fields could facilitate shared decision-making. Education, combined with targeted design, can help effectively implement digital health in cancer care.

Driven by the issue raised in our paper regarding the digital health literacy gap for patients with cancer, microlearning could help address the need to learn infrequently used or new skills, thereby improving patient safety [64]. A previous feasibility study of microlearning to deliver information on chemotherapy side effects to patients with lung cancer demonstrated improvements in participants’ cancer literacy and self-management coping strategies [73]. However, to our knowledge, our paper is the first to propose microlearning as a subscription model for designing a DHL intervention. Future work should focus on creating a prototype and examining its feasibility for short-term implementation.

Despite using microlearning as an instructional educational strategy, it may eventually be superseded by emerging innovations such as nanolearning, metaverse learning, or artificial intelligence–assisted learning, which promise opportunities to improve the quality and accessibility of education and to facilitate lifelong learning.

### Conclusion

Patients with cancer and caregivers were motivated to use modern technology-driven solutions to improve cancer DHL. The participants’ preferences regarding DHL training aligned with the innovative microlearning pedagogy. Microlearning could leverage technology to deliver tailored digital health literacy training for patients with cancer. Collaboration with multiple medical and nonmedical stakeholders could facilitate the delivery of cancer DHL training.

Future work should focus on designing and assessing the feasibility of implementing a microlearning-based DHL training program that involves end users and diverse stakeholders.

## Supplementary material

10.2196/77738 Multimedia Appendix 1 Arabic and English versions of the interview guide.

10.2196/77738 Checklist 1 COREQ (Consolidated Criteria for Reporting qualitative research) checklist.

## Abbreviations

COREQ: Consolidated Criteria for Reporting Qualitative Research
DHL: digital health literacy
KFSH: King Fahad Specialist Hospital
PILOT: purpose, information, links, originator, timeliness
SEEK: Source, Evidence, Explanation, and Knowledge
UTAUT: Unified Theory of Acceptance and Use of Technology

## References

[1] Vance K, Howe W, Dellavalle RP (2009). Social internet sites as a source of public health information. Dermatol Clin.

[2] Al-Kahtani N, Alruwaie S, Al-Zahrani BM (2022). Digital health transformation in Saudi Arabia: a cross-sectional analysis using Healthcare Information and Management Systems Society’ digital health indicators. Digit Health.

[3] Rahman R, Al-Borie HM (2021). Strengthening the Saudi Arabian healthcare system: role of Vision 2030. Int J Healthc Manag.

[4] AlMuammar SA, Noorsaeed AS, Alafif RA, Kamal YF, Daghistani GM (2021). The use of internet and social media for health information and its consequences among the population in Saudi Arabia. Cureus.

[5] Fujisawa D, Hagiwara N (2015). Cancer stigma and its health consequences. Curr Breast Cancer Rep.

[6] Al-Azri M, Al-Awisi H, Al-Rasbi S (2014). Psychosocial impact of breast cancer diagnosis among Omani women. Oman Med J.

[7] Fareed N, Swoboda CM, Jonnalagadda P, Huerta TR (2021). Persistent digital divide in health-related internet use among cancer survivors: findings from the Health Information National Trends Survey, 2003-2018. J Cancer Surviv.

[8] Penedo FJ, Natori A, Fleszar-Pavlovic SE (2023). Factors associated with unmet supportive care needs and emergency department visits and hospitalizations in ambulatory oncology. JAMA Netw Open.

[9] Yoo SH, Sung JH, Lee K (2024). The needs for digital health and eHeath literacy of cancer patients, caregivers, and healthcare providers: a multicenter, descriptive correlational study. Eur J Oncol Nurs.

[10] Sanchiz M, Amadieu F, Fu WT, Chevalier A (2019). Does pre-activating domain knowledge foster elaborated online information search strategies? Comparisons between young and old web user adults. Appl Ergon.

[11] Ahles TA, Root JC (2018). Cognitive effects of cancer and cancer treatments. Annu Rev Clin Psychol.

[12] Mohamed H, O’Malley L, Kelly D (2023). An infodemiology study on exploring the quality and reliability of colorectal cancer immunotherapy information. Digit Health.

[13] Almoajel A, Alshamrani S, Alyabsi M (2022). The relationship between e-Health literacy and breast cancer literacy among Saudi women. Front Public Health.

[14] Alhur A, Alhur A, Alshammari M (2023). Digital health literacy and web-based health information-seeking behaviors in the Saudi Arabian population. Cureus.

[15] Mackert M, Mabry-Flynn A, Champlin S, Donovan EE, Pounders K (2016). Health literacy and health information technology adoption: the potential for a new digital divide. J Med Internet Res.

[16] Zarcadoolas C, Vaughon WL, Czaja SJ, Levy J, Rockoff ML (2013). Consumers’ perceptions of patient-accessible electronic medical records. J Med Internet Res.

[17] Mohamed H, Kittle E, Nour N (2024). An integrative systematic review on interventions to improve layperson’s ability to identify trustworthy digital health information. PLOS Digit Health.

[18] Kemp E, Trigg J, Beatty L (2021). Health literacy, digital health literacy and the implementation of digital health technologies in cancer care: the need for a strategic approach. Health Promot J Austr.

[19] Rising KL, Guth A, Gentsch AT (2024). Development and preliminary validation of a screener for digital health readiness. JAMA Netw Open.

[20] Batalden M, Batalden P, Margolis P (2016). Coproduction of healthcare service. BMJ Qual Saf.

[21] McColl-Kennedy JR, Snyder H, Elg M (2017). The changing role of the health care customer: review, synthesis and research agenda. JOSM.

[22] Kim SH, Sung JH, Yoo SH (2023). Effects of digital self-management symptom interventions on symptom outcomes in adult cancer patients: a systematic review and meta-analysis. Eur J Oncol Nurs.

[23] Roberts AL, Fisher A, Smith L, Heinrich M, Potts HWW (2017). Digital health behaviour change interventions targeting physical activity and diet in cancer survivors: a systematic review and meta-analysis. J Cancer Surviv.

[24] Shaffer KM, Turner KL, Siwik C (2023). Digital health and telehealth in cancer care: a scoping review of reviews. Lancet Digit Health.

[25] Nutbeam D (2004). Getting evidence into policy and practice to address health inequalities. Health Promot Int.

[26] Eccles MP, Armstrong D, Baker R (2009). An implementation research agenda. Implementation Sci.

[27] Lee H, Oldewage-Theron W (2023). Process evaluation of a theory-informed eHealth intervention through “Healthy Online Parental Education (HOPE)” study. J Nutr Educ Behav.

[28] Venkatesh V, Morris MG, Davis GB, Davis FD (2003). User acceptance of information technology: toward a unified view. MIS Q.

[29] Scur G, da Silva AVD, Mattos CA, Gonçalves RF (2023). Analysis of IoT adoption for vegetable crop cultivation: multiple case studies. Technol Forecast Soc Change.

[30] Al-Sharafi MA, Al-Emran M, Arpaci I (2023). Generation Z use of artificial intelligence products and its impact on environmental sustainability: a cross-cultural comparison. Comput Human Behav.

[31] Gu D, Khan S, Khan IU (2021). Assessing the adoption of e-Health technology in a developing country: an extension of the UTAUT model. Sage Open.

[32] Cobelli N, Cassia F, Donvito R (2023). Pharmacists’ attitudes and intention to adopt telemedicine: integrating the market-orientation paradigm and the UTAUT. Technol Forecast Soc Change.

[33] Tong A, Sainsbury P, Craig J (2007). Consolidated criteria for reporting qualitative research (COREQ): a 32-item checklist for interviews and focus groups. Int J Qual Health Care.

[34] Downe-Wamboldt B (1992). Content analysis: method, applications, and issues. Health Care Women Int.

[35] DeJonckheere M, Vaughn LM (2019). Semistructured interviewing in primary care research: a balance of relationship and rigour. Fam Med Community Health.

[36] Palinkas LA, Horwitz SM, Green CA, Wisdom JP, Duan N, Hoagwood K (2015). Purposeful sampling for qualitative data collection and analysis in mixed method implementation research. Adm Policy Ment Health.

[37] Hennink MM, Kaiser BN, Marconi VC (2017). Code saturation versus meaning saturation: how many interviews are enough?. Qual Health Res.

[38] Naeem M, Ozuem W, Howell K, Ranfagni S (2023). A step-by-step process of thematic analysis to develop a conceptual model in qualitative research. Int J Qual Methods.

[39] Giles EL, Harrison SL (2023). Reflecting on the importance of theory-informed qualitative research in people with chronic respiratory disease and their carers. Chron Respir Dis.

[40] Gale NK, Heath G, Cameron E, Rashid S, Redwood S (2013). Using the framework method for the analysis of qualitative data in multi-disciplinary health research. BMC Med Res Methodol.

[41] O’Connor C, Joffe H (2020). Intercoder reliability in qualitative research: debates and practical guidelines. Int J Qual Methods.

[42] McKim C (2023). Meaningful member-checking: a structured approach to member-checking. Am J Qualitative Res.

[43] Bahkali S, Alfurih S, Aldremly M, Alzayyat M, Alsurimi K, Househ M (2016). The prevalence of internet and social media based medication information seeking behavior in Saudi Arabia. Stud Health Technol Inform.

[44] Alhur A (2022). Exploring Saudi Arabia individuals’ attitudes toward electronic personal health records. JCSTS.

[45] Marouf A, Tayeb R, Alshehri GD (2023). Public perception of common cancer misconceptions: a nationwide cross-sectional survey and analysis of over 3500 participants in Saudi Arabia. J Family Med Prim Care.

[46] Car J, Lang B, Colledge A, Ung C, Majeed A (2011). Interventions for enhancing consumers’ online health literacy. Cochrane Database Syst Rev.

[47] Mead N, Varnam R, Rogers A, Roland M (2003). What predicts patients’ interest in the Internet as a health resource in primary care in England?. J Health Serv Res Policy.

[48] Gray NJ, Klein JD, Noyce PR, Sesselberg TS, Cantrill JA (2005). The internet: a window on adolescent health literacy. J Adolesc Health.

[49] Austvoll-Dahlgren A, Bjørndal A, Odgaard-Jensen J, Helseth S (2012). Evaluation of a web portal for improving public access to evidence-based health information and health literacy skills: a pragmatic trial. PLoS ONE.

[50] Pennycook G, McPhetres J, Zhang Y, Lu JG, Rand DG (2020). Fighting COVID-19 misinformation on social media: experimental evidence for a scalable accuracy-nudge intervention. Psychol Sci.

[51] Mörelius E, Robinson S, Arabiat D, Whitehead L (2021). Digital interventions to improve health literacy among parents of children aged 0 to 12 years with a health condition: systematic review. J Med Internet Res.

[52] Xie B (2012). Improving older adults’ e-health literacy through computer training using NIH online resources. Libr Inf Sci Res.

[53] Evans P, Aungst TD, Massey C, Bartlett D (2015). Expanding clinical and information services to the ambulatory older adult through community outreach programs. Consult Pharm.

[54] Sankaranarayanan R, Leung J, Abramenka-Lachheb V, Seo G, Lachheb A (2023). Microlearning in diverse contexts: a bibliometric analysis. TechTrends.

[55] Alsaad E, AlDossary S (2024). Educational video intervention to improve health misinformation identification on WhatsApp among Saudi Arabian population: pre-post intervention study. JMIR Form Res.

[56] Nokes KM, Reyes DM (2020). Internet use for health-related information: self-care agency of lower income persons living with HIV/AIDS. Nurs Sci Q.

[57] Kim H, Goldsmith JV, Sengupta S (2019). Mobile health application and e-Health literacy: opportunities and concerns for cancer patients and caregivers. J Canc Educ.

[58] Mosa ASM, Yoo I, Sheets L (2012). A systematic review of healthcare applications for smartphones. BMC Med Inform Decis Mak.

[59] Muscat DM, Lambert K, Shepherd H (2021). Supporting patients to be involved in decisions about their health and care: development of a best practice health literacy app for Australian adults living with chronic kidney disease. Health Prom J of Aust.

[60] Ruland CM, Jeneson A, Andersen T (2007). Designing tailored Internet support to assist cancer patients in illness management. AMIA Annu Symp Proc.

[61] Román-Sánchez D, De-La-Fuente-Rodríguez JM, Paramio A, Paramio-Cuevas JC, Lepiani-Díaz I, López-Millan MR (2023). Evaluating satisfaction with teaching innovation, its relationship to academic performance and the application of a video-based microlearning. Nurs Open.

[62] Flornoy-Guédon A, Fonzo-Christe C, Meier E (2024). Development and evaluation of a blended learning training programme for pharmacy technicians’ continuing education. Eur J Hosp Pharm.

[63] Zolfaghari M, Shirzadi S, Motamed M (2023). Using a mobile application for psychiatry training in medical students: a quasi-experimental study. Australas Psychiatry.

[64] Buchem I, Hamelmann H (2010). Microlearning: a strategy for ongoing professional development. Microcontent and Microlearning.

[65] Carter JW, Youssef-Morgan C (2022). Psychological capital development effectiveness of face-to-face, online, and micro-learning interventions. Educ Inf Technol (Dordr).

[66] Taylor A, Hung W (2022). The effects of microlearning: a scoping review. Education Tech Research Dev.

[67] Sun G, Cui T, Yong J, Shen J, Chen S (2018). MLaaS: a cloud-based system for delivering adaptive micro learning in mobile MOOC learning. IEEE Trans Serv Comput.

[68] Hoffman-Goetz L, Friedman DB, Celestine A (2006). Evaluation of a public library workshop: Teaching older adults how to search the internet for reliable cancer information. J Consum Health Internet.

[69] De Main AS, Xie B, Shiroma K, Yeh T, Davis N, Han X (2022). Assessing the effects of eHealth tutorials on older adults’ eHealth literacy. J Appl Gerontol.

[70] Kammerer Y, Amann DG, Gerjets P (2015). When adults without university education search the internet for health information: the roles of Internet-specific epistemic beliefs and a source evaluation intervention. Comput Human Behav.

[71] Kalichman SC, Cherry C, Cain D (2006). Internet-based health information consumer skills intervention for people living with HIV/AIDS. J Consult Clin Psychol.

[72] Bernstam EV, Walji MF, Sagaram S, Sagaram D, Johnson CW, Meric-Bernstam F (2008). Commonly cited website quality criteria are not effective at identifying inaccurate online information about breast cancer. Cancer.

[73] Janssen A, Shah K, Rabbets M (2023). Feasibility of microlearning for improving the self-efficacy of cancer patients managing side effects of chemotherapy. J Cancer Educ.

[74] Gray K, Elliott K, Wale J (2013). A community education initiative to improve using online health information: participation and impact. Inform Health Soc Care.

[75] Xie B (2011). Older adults, e-health literacy, and collaborative learning: an experimental study. J Am Soc Inf Sci.

[76] Lee K, Hoti K, Hughes JD, Emmerton LM (2014). Interventions to assist health consumers to find reliable online health information: a comprehensive review. PLoS ONE.

[77] Emerson LC, Berge ZL (2018). Microlearning: knowledge management applications and competency-based training in the workplace. Knowledge Management & E-Learning.

[78] Henderson S, Wagner JL, Gosdin MM (2020). Complexity in partnerships: a qualitative examination of collaborative depression care in primary care clinics and community-based organisations in California, United States. Health Soc Care Community.

[79] Harris JR, Wallace RB (2012). The Institute of Medicine’s new report on living well with chronic illness. Prev Chronic Dis.

[80] Miranda J, Ong MK, Jones L (2013). Community-partnered evaluation of depression services for clients of community-based agencies in under-resourced communities in Los Angeles. J GEN INTERN MED.

[81] Predmore Z, Hatef E, Weiner JP (2019). Integrating social and behavioral determinants of health into population health analytics: a conceptual framework and suggested road map. Popul Health Manag.

[82] Morley J, Cowls J, Taddeo M, Floridi L (2020). Public health in the information age: recognizing the infosphere as a social determinant of health. J Med Internet Res.

