# Supplementary material for: Exploring the Readiness for Digital Health Literacy Transformation and Intervention Preferences From the Perspectives of Patients With Cancer, Caregivers, and Health Care Professionals: Qualitative Interview Study
Source: JMIR Cancer. 2026 Mar 4;12:e77738. doi: 10.2196/77738 (PMC12977330; doi:10.2196/77738)
Supplement: Multimedia Appendix 1 [file cancer-v12-e77738-s001.docx]

**Interview meeting topic guide for cancer patients, caregivers, and healthcare professionals (English version)**

**Digital learnability**

1.How do you describe your/ patients’ ability to use digital technology?

2. How do you believe about your/patients’ interest to engage with new technology?

**Digital health literacy training preferences**

**DHL training mode of delivery**

3. What location (setting) suits you/patients to get the digital health literacy training?

4. What mode of delivery works better for you/patients to get the digital health literacy training?

**DHL training information content/information display?**

5. What specific topics you would like to see covered in such training?

6. What is the preferred mode to present the information in the digital health literacy training?

**DHL training interval/duration/language**

7. What training style works better for you to get the digital health literacy training?

8. What do you think about the duration of the digital health literacy training?

9. What is the preferred language to get the digital health literacy training?

**DHL training design**

10. What do you suggest for designing the digital health literacy training?

**Facilitators of the DHL training adoption**

11. What would facilitate the delivery the digital health literacy training?

**Interview meeting topic guide for cancer patients, caregivers, and healthcare professionals (Arabic version)**

**دليل موضوعات اجتماع المقابلة لمرضى السرطان ومقدمي الرعاية والمهنيين الصحيين**

**قابلية التعلم الرقمي**

١. كيف تصف قدرتك/قدرة المرضى على استخدام التكنولوجيا الرقمية؟

٢. ما رأيك في اهتمامك/اهتمام المرضى بالانخراط في التكنولوجيا الجديدة؟

**تفضيلات التدريب على الثقافة الصحية الرقمية**

**أسلوب تقديم تدريب الثقافة الصحية الرقمية**

٣. ما المكان (البيئة) الذي يناسبك للحصول على تدريب الثقافة الصحية الرقمية؟

٤. ما أسلوب تقديم التدريب الذي يعمل بشكل أفضل بالنسبة لك للحصول على تدريب الثقافة الصحية الرقمية؟

**محتوى المعلومات/عرض المعلومات في التدريب**

٥. ما الموضوعات المحددة التي ترغب في تضمينها في مثل هذا التدريب؟

٦. ما الطريقة المفضلة لديك لعرض المعلومات في تدريب الثقافة الصحية الرقمية؟

**فترة التدريب/مدته/اللغة**

٧. ما أسلوب التدريب الذي يناسبك بشكل أفضل للحصول على تدريب الثقافة الصحية الرقمية؟

٨. ما رأيك في مدة تدريب الثقافة الصحية الرقمية؟

٩. ما اللغة المفضلة للحصول على تدريب الثقافة الصحية الرقمية؟

**تصميم التدريب**

١٠. ما اقتراحاتك لتصميم تدريب الثقافة الصحية الرقمية؟

**العوامل الميسّرة لاعتماد التدريب**

١١. ما الذي يمكن أن يسهل تقديم تدريب الثقافة الصحية الرقمية؟
